# NEU3 at the plasma membrane: context-dependent ganglioside remodeling in health and disease

**DOI:** 10.1016/j.jlr.2026.101175

**Published:** 2026-10-21

**Authors:** Federica Cirillo, Paola Rota, Luigi Anastasia, Marco Piccoli

**Affiliations:** 1 Laboratory of Stem Cells for Tissue Engineering, IRCCS Policlinico San Donato, Milan, Italy; 2 Institute for Molecular and Translational Cardiology (IMTC), IRCCS Policlinico San Donato, Milan, Italy; 3 Department of Biomedical, Surgical and Dental Sciences, University of Milan, Milan, Italy; 4 Vita-Salute San Raffaele University, Milan, Italy

**Keywords:** cancer, cell signaling, fibrosis, glycosphingolipids, Sialidases, Sphingolipids, Small molecules

## Abstract

Cell-surface gangliosides tune receptor signaling, cell-state transitions, and responses to stress. NEU3 is a mammalian sialidase associated with the plasma membrane and endomembranes that preferentially removes terminal sialic acids from gangliosides. Here, we critically review biochemical studies, cultured-cell experiments, animal models, and observations in human tissues that link NEU3 to myogenesis, hypoxic adaptation, cardioprotection, ganglioside storage disorders, cancer, fibrosis, and intestinal inflammation. Across these settings, NEU3 can alter the local ganglioside environment of receptors and signaling complexes, but the strength and direction of the resulting effects vary with cell type, substrate availability, localization, expression level, and duration of activity. We therefore frame NEU3 as a context-dependent regulator rather than a uniformly beneficial or harmful enzyme. This evidence-weighted model helps reconcile apparently opposing findings while highlighting important uncertainties in membrane topology, extracellular delivery, substrate selection, and pharmacological specificity. Selective inhibitors, tissue-restricted delivery, and biomarker-guided approaches are promising, but clinical translation will require stronger target-engagement data and broader in vivo validation.

The eukaryotic cell is surrounded by a dense layer of oligosaccharides, the glycocalyx: not a passive barrier but a dynamic interface mediating interactions with the environment, neighboring cells, and the extracellular matrix. At the termini of these glycoconjugates are sialic acids, a family of nine-carbon α-keto acids (nonulosonic acids) that carry a strong negative charge at physiological pH and are principal determinants of the cell’s molecular topography (1).

Sialic acids mask antigenic sites, regulate the half-life of circulating glycoproteins, mediate cell–cell adhesion through selectins and Siglecs, and modulate transmembrane receptors 1, 2. As self-associated molecular patterns, they also engage inhibitory Siglecs and help restrain complement and natural-killer-cell responses (3). In this review, the term rheostat denotes the reversible, graded control of membrane-proximal signaling produced by the opposing actions of Golgi sialyltransferases and compartment-specific sialidases 4, 5.

Sialidases were historically viewed as lysosomal enzymes dedicated to sequential glycoconjugate catabolism. Indeed, deficiency of the lysosomal sialidase NEU1 causes sialidosis (mucolipidosis type I), a severe storage disorder marked by accumulation of sialylated oligosaccharides (6). However, the cloning of four distinct mammalian isoenzymes, NEU1, NEU2, NEU3, and NEU4, in the late 1990s and early 2000s overturned this purely catabolic paradigm 7, 8, 9, 10, 11. These enzymes show distinct subcellular localizations, pH optima, substrate specificities, and tissue expression, pointing to specialized, non-redundant roles in cellular signaling (12).

We write NEU3 for the human enzyme and Neu3 for the murine ortholog, following the source of each finding; where a human enzyme was expressed in a rodent or primate cell system, we name the enzyme and the system separately. Among the four mammalian enzymes, NEU3 is distinguished by its strong association with the plasma membrane and endomembranes and by preferential, although not exclusive, activity toward gangliosides 10, 12. Its proximity to lipid rafts, caveolae, and receptor complexes provides a plausible route by which ganglioside remodeling can influence receptor-proximal signaling. The receptor systems in which this has been examined, EGFR, the insulin receptor, β1 integrin, and the Wnt co-receptor LRP6, are considered in turn below.

NEU3 has often been described as “Janus-faced” because increased activity can support differentiation or stress adaptation in one setting yet accompany cancer, fibrosis, or inflammation in another. We use this metaphor only as an organizing device and evaluate each claim according to the underlying evidence: direct biochemistry, cultured-cell perturbation, causal animal experiments, or observations in human tissue. The result is a context-dependent model rather than a binary classification of NEU3 as beneficial or harmful (Fig. 1A).

**Fig. 1:**
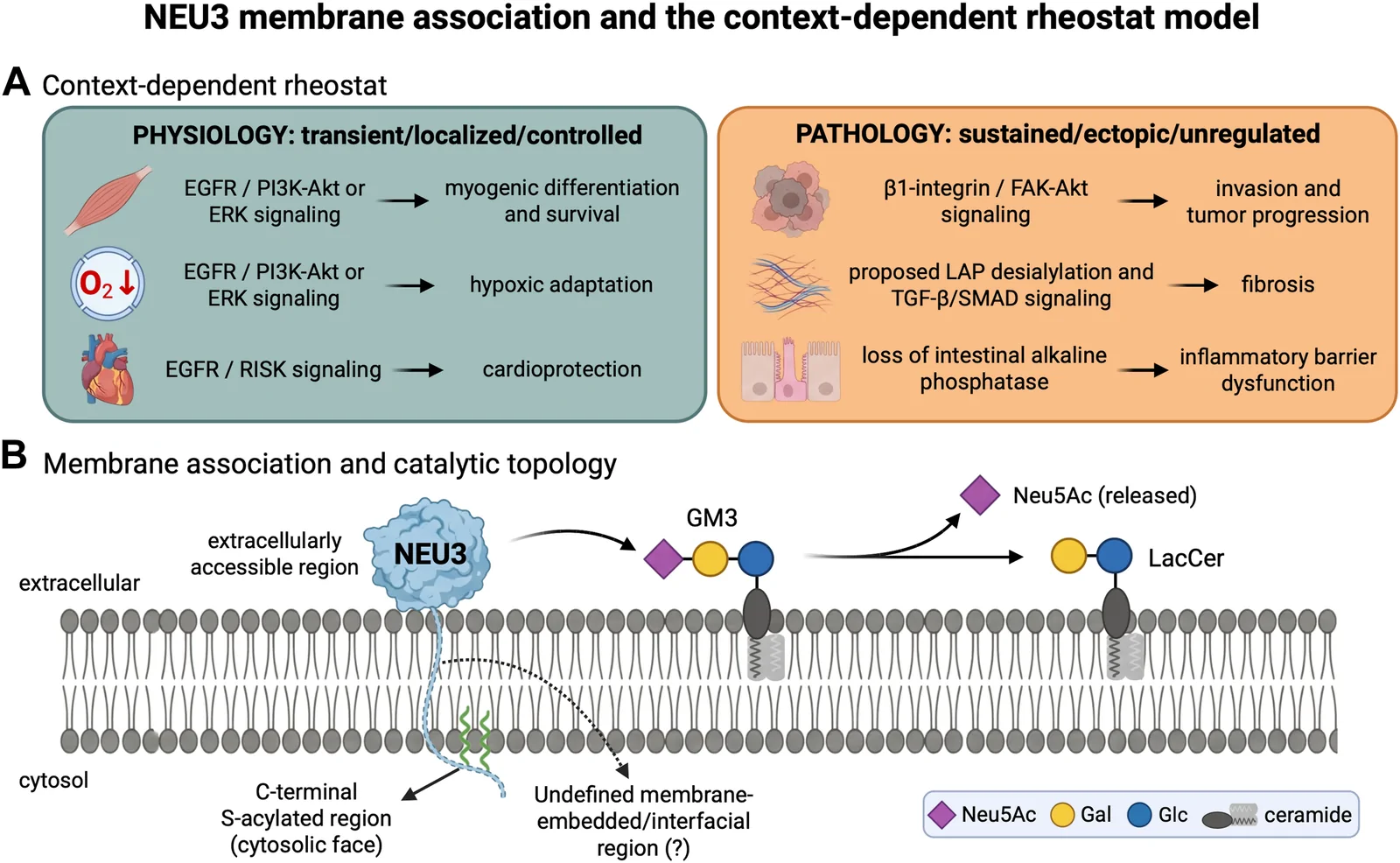
NEU3 membrane association and the context-dependent rheostat model. A, context-dependent rheostat. The same enzymatic activity is associated with opposite outcomes depending on context. Transient, localized and controlled activity accompanies EGFR/PI3K–Akt or ERK signaling in myogenic differentiation and survival, the same signaling axes in hypoxic adaptation, and EGFR/RISK signaling in cardioprotection. Sustained, ectopic or unregulated activity accompanies β1 integrin/FAK–Akt signaling in invasion and tumor progression, the proposed desialylation of latency-associated peptide with downstream TGF-β/SMAD signaling in fibrosis, and loss of intestinal alkaline phosphatase with inflammatory barrier dysfunction. The panel is an organizing framework for sections 3–5 and does not imply equivalence of evidence between the two columns. B, membrane association and catalytic topology. NEU3 lacks a canonical α-helical transmembrane segment, and the two studies that tested its membrane association directly disagree on whether it behaves as a peripheral or as an integral membrane protein 13, 14; the panel therefore shows membrane association without asserting a resolved topology. An extracellularly accessible portion of the protein is drawn above the outer leaflet, whereas the cytosolic C-terminal region is shown as a dashed unstructured segment; the S-acyl chains (green) are placed there because S-acylation occurs only on the cytosolic face, although the modified cysteines have not been mapped. The portion that traverses the bilayer is labeled explicitly as an undefined membrane-embedded or interfacial region, because no canonical α-helical transmembrane segment is supported by current predictors and NEU3 has no signal peptide. For drawing purposes the panel is rendered after the integral-like model, because the features drawn, an extracellularly accessible portion, a cytosolic C-terminal region and cytosolic S-acylation, are the ones that model specifies; this is a drawing convention rather than a judgement between the three readings, and neither the peripheral outer-leaflet model nor the type I orientation is drawn. The ceramide anchor of the ganglioside is confined to the outer leaflet. NEU3 removes only the terminal Neu5Ac from GM3, releasing free Neu5Ac and leaving LacCer; the oligosaccharide is not cleaved as a block, and the released sialic acid does not bind a receptor. Glycans follow the SNFG: purple diamond, Neu5Ac; yellow circle, Gal; blue circle, Glc; gray, ceramide, with the sphingoid base and the N-acyl chain drawn differently. The panel does not assign the catalytic site to a particular portion of the protein: the reaction is drawn at the outer leaflet because that is where the ganglioside substrate resides, not because the orientation of the active site has been determined. How the protein engages the bilayer also remains unresolved, and panel B is a working model, not a solved structure. Akt, protein kinase B; EGFR, epidermal growth factor receptor; ERK, extracellular signal-regulated kinase; FAK, focal adhesion kinase; GM3, monosialodihexosylganglioside; LacCer, lactosylceramide; Neu5Ac, N-acetylneuraminic acid; PI3K, phosphoinositide 3-kinase; RISK, reperfusion injury salvage kinase; SMAD, small mothers against decapentaplegic homolog; TGF-β, transforming growth factor β; SNFG, Symbol Nomenclature for Glycans Created in BioRender. Foglio, E. (2026) https://BioRender.com/80hf8ej.

Literature approach: we searched PubMed with the query NEU3[Title] OR Neu3[Title] OR “sialidase 3”[Title] OR “neuraminidase 3”[Title], restricted to January 2020 through August 2026, and screened cited primary studies relevant to the review’s scope. Throughout the review, we distinguish direct biochemical evidence, cellular association, causal in vivo data, and translational hypotheses.

## Biochemical basis of NEU3 action

The four mammalian sialidases are predicted to share a conserved six-bladed β-propeller catalytic fold, verified experimentally only for NEU2, but differ in localization, substrate preference, and biological function (Table 1) 12, 15. NEU1 is mainly lysosomal and depends on PPCA; NEU2 is a low-abundance cytosolic enzyme; and NEU4 comprises isoforms associated with mitochondria, the endoplasmic reticulum, and lysosomes 6, 12, 16, 17, 18, 19, 20, 21, 22, 23, 24, 25, 26, 27. These distinctions limit functional extrapolation from one isoenzyme to another.

**Table 1:** Comparative properties of the four mammalian sialidases

Isoenzyme	Subcellular Localization	Key Substrates	Physiological and Pathological Roles
NEU1	Primarily lysosomal lumen; translocates to plasma membrane in immune cells	Sialylated oligosaccharides and glycopeptides in the lysosome; TLR4 and the insulin receptor proposed at the plasma membrane	Modulates innate immune response (TLR4 signaling) and acts as a metabolic gatekeeper for insulin signaling
NEU2	Cytosol	Broad specificity: glycoproteins and gangliosides	Involved in intracellular remodeling during myogenesis
NEU3	Plasma membrane-associated, and also present on endosomal and lysosomal membranes; enriched in lipid rafts and caveolae. Mode of membrane association disputed: reported as a peripheral protein of the outer leaflet; as a type I protein with an extracytoplasmic N terminus, from protease protection of the bovine enzyme; and as behaving like an integral membrane protein, S-acylated with a cytosolic C terminus and an extracellularly accessible portion. No canonical α-helical transmembrane segment is supported by current predictors; exact topology unresolved	Strongest preference for gangliosides (GM3, GD1a)	Implicated in myoblast differentiation and in survival under hypoxic stress; cardioprotective effects reported in preclinical models. Involved in tumor progression, metastasis, tissue fibrosis (proposed activation of latent TGF-β1), and intestinal inflammation
NEU4	Isoform-dependent: mitochondria (long form), endoplasmic reticulum and lysosomes (short form)	NCAM, mucins/O-glycans, and gangliosides	Regulates neuronal plasticity, CNS development, and brain homeostasis; acts on mucin O-glycans and has been linked to colon cancer

CNS, central nervous system; GD1a and GM3, ganglioside species (see Fig. 1); NCAM, neural cell adhesion molecule; NEU, neuraminidase/sialidase; TGF-β1, transforming growth factor β1; TLR4, Toll-like receptor 4. How NEU3 associates with the bilayer is disputed between the primary studies, and its exact topology is not resolved; the NEU3-dependent activation of latent TGF-β1 is a proposed cellular mechanism. Both points are discussed in the text.

NEU3 is the principal focus here because it preferentially hydrolyzes gangliosides, is tightly associated with cellular membranes, and can act on substrates in the same membrane or, under selected experimental conditions, on neighboring membranes 10, 28, 29. These properties place it at the interface between lipid metabolism and receptor-proximal signaling.

### Gene regulation, membrane topology, and trafficking

Human NEU3 was identified in 2000 from an expressed-sequence-tag clone and shown to encode a 428-residue, approximately 48 kDa protein carrying the YRIP sequence motif and three Asp boxes, the aspartate-rich repeats characteristic of sialidase β-propellers; it maps to chromosome 11q13.5 (11). The orthologous enzyme had been purified from bovine brain and cloned shortly before 10, 30. Distinguished by plasma membrane localization and strong activity toward gangliosides such as GM3 and GD1a, NEU3 is highly conserved: the human protein shares 78% sequence identity with the bovine enzyme (11), and the murine ortholog (Neu3) has been characterized separately (28).

Transcription of NEU3 is tightly and tissue-specifically regulated. Reported tissue distribution depends on the method: Northern analysis of human NEU3 showed a wide pattern of expression in adult and fetal tissues (11), whereas later surveys describe relative enrichment in adrenal gland, skeletal muscle, heart, liver, and colon (12). Chronic inflammation and malignancy trigger upregulation via several mechanisms:

Hypoxia increases NEU3 transcription in the experimental systems examined, but the promoter does not appear to be a direct HIF-1α target. The gene instead has alternative promoters with two clusters of transcription start sites; the region lacks a TATA box and contains CCAAT and CAAC elements whose deletion lowers promoter activity, and Sp1 and Sp3 bind it and appear to govern start-site selection rather than acting as simple activators, since their knockdown raises transcription from brain-type start sites while lowering it from others (31). Hypoxic induction of NEU3 was linked to Sp1/Sp3 separately, in C2C12 cells (32); the promoter study itself contains no hypoxia experiment. Consistent with a role for Sp-family factors, SP3 knockdown lowers NEU3 in TNF-α-stimulated human endothelial cells, and NEU3 is raised in atherosclerotic plaque, although SP3 itself was not measured in that tissue (33). General links between hypoxia, HIF-1, and Sp1 provide context for this regulatory architecture but do not independently establish NEU3 regulation (34).

NEU3 expression and ganglioside-directed sialidase activity are elevated in several tumor models and in selected human cancers, particularly colorectal cancer 31, 35. Hypoxia, oxidative stress, and oncogenic signaling are plausible contributors, but the relative importance of promoter occupancy, epigenetic state, and gene dosage remains incompletely resolved.

Inflammatory signaling may also participate in positive feedback. Recombinant NEU3 can stimulate IL-6 and IL-1β release from peripheral blood mononuclear cells, whereas IL-6 increases NEU3 abundance in monocytes and lymphocytes 36, 37. These observations support amplification of inflammatory responses, although the relevant cell source, localization, and catalytic target in vivo require further definition.

NEU3 is predicted to retain the six-bladed β-propeller fold characteristic of mammalian sialidases. No experimental structure of human NEU3 has yet been solved; current models are therefore useful hypotheses for residue placement and ligand recognition rather than substitutes for structural validation (15).

The catalytic site lies in a central crevice with a conserved arginine triad anchoring the sialic acid carboxylate. Cleavage follows a double-displacement, net-retaining mechanism: Tyr370, activated by Glu225, attacks the anomeric center to form a covalent sialyl-enzyme intermediate, while Asp50 acts as the general acid/base. These assignments derive from homology modeling on the NEU2 structure combined with site-directed mutagenesis of human NEU3, not from an experimental NEU3 structure 12, 15, 38.

How NEU3 associates with membranes is disputed, and the primary studies do not agree. Zanchetti *et al.* solubilized the enzyme under conditions that release peripheral membrane proteins and found it hydrophilic by Triton X-114 partitioning, concluding that NEU3 is a peripheral protein of the outer leaflet (13). Rodriguez-Walker and Daniotti found that NEU3 is not released under those conditions, that its C terminus faces the cytosol while another portion is accessible from the extracellular space, and that the enzyme is S-acylated, a modification confined to the cytosolic face, although the modified cysteines were not mapped, concluding that it behaves like an integral membrane protein (14). The original cloning reports predicted a putative transmembrane segment from sequence alone 10, 11, and a protease-protection experiment on the bovine enzyme suggested a type I orientation with an extracytoplasmic N terminus and no signal sequence (10); the later study explicitly excludes a type I arrangement, finds no canonical α-helical transmembrane segment by several independent predictors, and places the C terminus, and probably the N terminus as well, on the cytosolic face (14). Three primary studies therefore bear on the question and reach incompatible conclusions. We present this disagreement rather than resolve it. How the polypeptide crosses or engages the bilayer, the orientation of the catalytic site, and the export route all remain unresolved, and Figure 1B should be read accordingly as a working model.

The mouse enzyme hydrolyzes gangliosides in cis and, in mixed culture, acts on gangliosides presented at the surface of neighboring cells; in that system trans activity was the dominant mode rather than a marginal one (28). NEU3 has also been detected in extracellular vesicle-associated material (39). Activated microglia secrete Neu3 in association with fusogenic extracellular vesicles, and this secreted pool is both necessary and sufficient to desialylate neighboring neurons (40); vesicular release therefore offers one plausible route by which an enzyme lacking a signal peptide can reach the extracellular space. The abundance, orientation, and physiological range of extracellular activity remain uncertain, so these observations should not be interpreted as evidence for unrestricted soluble-enzyme action.

Association with caveolin-1 and enrichment in cholesterol-rich membrane domains can position NEU3 near receptors and signaling complexes 5, 41. NEU3 is also found on endosomal and lysosomal membranes 10, 13, 42. Defining the trafficking steps and post-translational modifications that control these pools is an important unresolved problem.

### Substrates and receptor-proximal signaling

In vitro, NEU3 preferentially hydrolyzes gangliosides over small soluble substrates and sialoglycoproteins 10, 30. Relevant reactions include GM3 to LacCer and GD1a to GM1a 10, 28; further conversion of GM1a to GA1 occurs in vivo in mouse models of GM1 gangliosidosis and in transduced patient fibroblasts, in which mouse NEU3 is approximately twofold more efficient than human NEU3 at this step (43). GM1a and GM2 are, however, poor substrates in the classical detergent-based in vitro assay (10), so the conversions relevant to storage disorders are established in cellular and animal contexts rather than predicted from the in vitro substrate profile. Reaction direction and product abundance therefore depend on the starting ganglioside repertoire, membrane organization, and duration of exposure.

Gangliosides are glycosphingolipids with a hydrophobic ceramide backbone and a sialic acid-bearing oligosaccharide headgroup, built stepwise by Golgi glycosyltransferases. At the surface they reside in the outer leaflet, enriched in the nervous system and in lipid rafts 44, 45, 46. LacCer is the common precursor of the ganglio-series. α2,3-Sialylation of its galactose by ST3GAL5 (GM3 synthase) gives GM3; α2,8-sialylation of the GM3 sialic acid by ST8SIA1 (GD3 synthase) gives GD3, and a further α2,8 transfer onto the terminal sialic acid of GD3 gives GT3. LacCer, GM3, GD3, and GT3 initiate the 0-, a-, b-, and c-series, respectively, and all four are elongated by the same branch enzymes, B4GALNT1 (GM2/GD2/GA2 synthase) and B3GALT4 (GM1a/GD1b/GA1 synthase); α2,3-sialylation of the terminal galactose by ST3GAL2 or ST3GAL3 then converts GM1a to GD1a and GD1b to GT1b 44, 45.

Gangliosides can modulate receptor tyrosine kinases through changes in membrane order and through specific lateral contacts. GM3 has been proposed to inhibit EGFR through carbohydrate-to-carbohydrate interactions involving receptor N-glycans 47, 48, while reconstituted-membrane studies also support direct lipid–protein and lipid-mediated conformational effects (49). These mechanisms are complementary rather than mutually exclusive.

By converting GM3 to LacCer, NEU3 can lower a ganglioside-dependent brake on EGFR. The enzyme should therefore be viewed as changing the probability or threshold of ligand-responsive receptor activation, not as transferring an oligosaccharide to EGFR or necessarily triggering ligand-independent signaling (50). The net effect depends on receptor abundance, ligand availability, local lipids, cell type, and exposure time.

## Physiological and adaptive roles

Neu3-null mice are viable and show comparatively mild baseline phenotypes, consistent with compensation or limited dependence under standard conditions; the same animals develop fewer colitis-associated colon adenocarcinomas than wild-type mice, while showing no difference in a chemically induced sporadic colon-cancer model (51). More informative phenotypes emerge during developmental transitions, hypoxia, ischemia–reperfusion, or ganglioside storage, where cell and animal studies reveal roles that remain strongly dependent on tissue and experimental context.

### Skeletal muscle

Myogenesis proceeds through an ordered sequence: myoblast proliferation, cell-cycle exit, expression of muscle-specific transcription factors (e.g. MyoD, Myogenin, MRF4), and alignment and fusion into multinucleated myotubes, accompanied by stage-specific changes in plasma membrane glycosphingolipids and ganglioside levels (50).

In C2C12 myoblasts, NEU3 abundance and plasma-membrane GM3 change during differentiation. Transient NEU3 upregulation during early commitment lowers GM3 and helps preserve the EGFR-dependent survival signaling needed before terminal differentiation (50).

NEU3 silencing increases GM3, attenuates EGFR–PI3K/Akt and ERK signaling, blocks myotube formation, and increases apoptosis (50). Thus, the inhibitory effect of silencing on the myoblast-to-myotube transition and the separate increase in cell death should be represented as related but distinct outcomes (Fig. 2A).

**Fig. 2:**
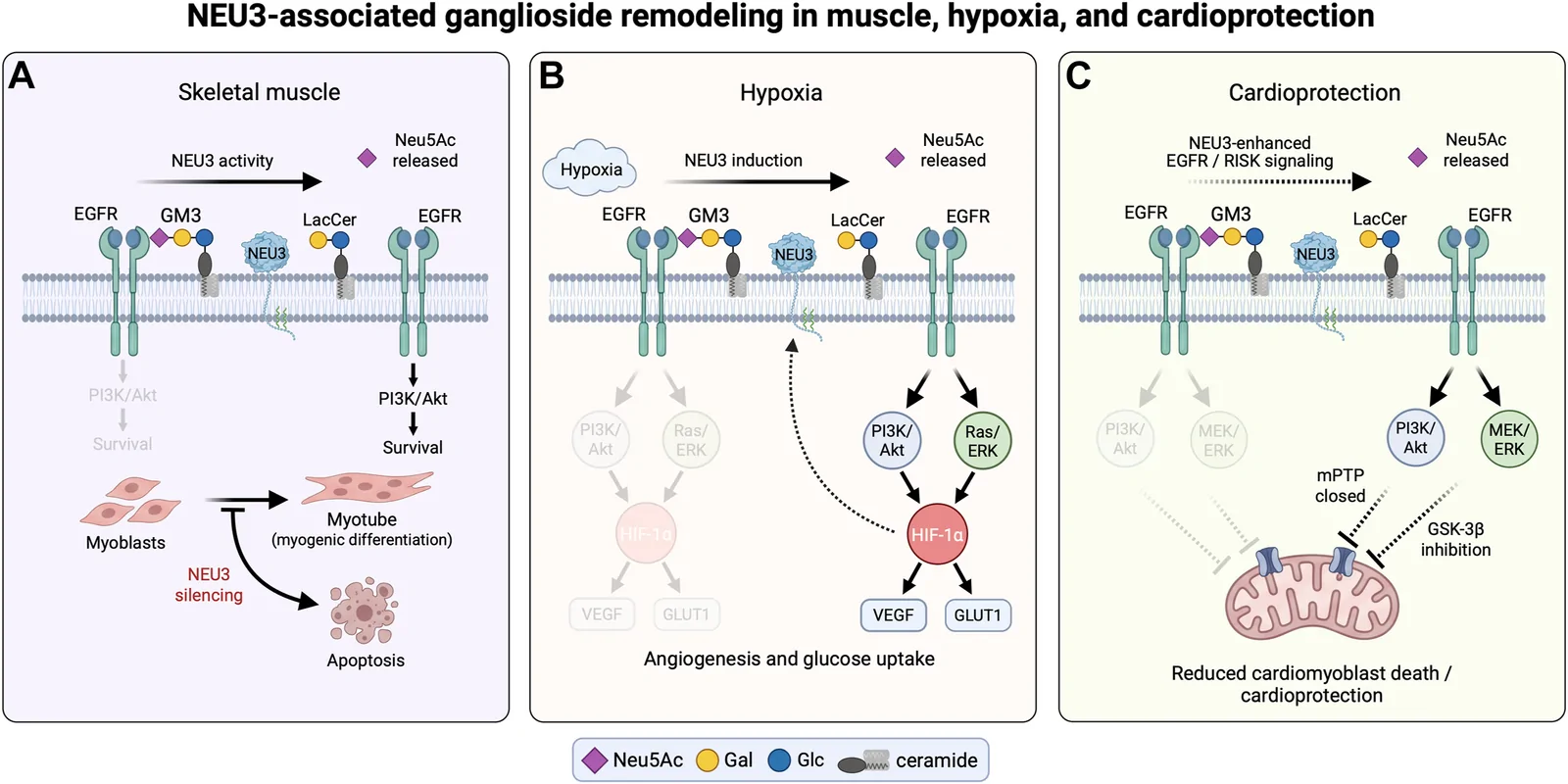
NEU3-associated ganglioside remodeling in muscle, hypoxia, and cardioprotection. The three panels use one representation: GM3 (Neu5Ac–Gal–Glc–ceramide) is converted to LacCer (Gal–Glc–ceramide) with release of free Neu5Ac; NEU3 is drawn with the same non-canonical membrane association as in Figure 1, its S-acylated region on the cytosolic face; and ceramide anchors occupy the outer leaflet only. Grayed elements denote the low-signaling state before ganglioside remodeling and are not separate pathways. A, skeletal muscle. While GM3 is present, EGFR-dependent PI3K/Akt signaling and survival are weak; after conversion to LacCer the same receptor supports survival signaling. NEU3 silencing is drawn as an inhibitory bar on the myoblast-to-myotube transition and as a separate arrow to apoptosis, because impaired differentiation and increased cell death are related but distinct outcomes. B, Hypoxia. NEU3 induction shifts the same receptor from weak to active PI3K/Akt and Ras/ERK signaling, reinforcing HIF-1α and its targets VEGF and GLUT1, and thereby angiogenesis and glucose uptake. A dashed arrow returns from HIF-1α to NEU3 to mark the proposed feed-forward step. Upstream of NEU3 induction the panel shows only the hypoxic stimulus: the Sp1/Sp3-dependent transcriptional step, and the evidence that the NEU3 promoter is not a direct HIF-1α target, are given in the text. C, Cardioprotection. What the cardiomyoblast study measured was NEU3 activity, Akt and ERK phosphorylation, HIF-1α and cell death; of these, Akt/ERK signaling and reduced cardiomyoblast death are drawn with solid arrows, and HIF-1α is not depicted in this panel because it is already shown in panel B. Dotted connectors mark the steps that were not assayed in that experiment and are carried here as established RISK biology: NEU3-enhanced EGFR/RISK signaling, GSK-3β inhibition, and closure of the mPTP. The GM3-to-LacCer conversion is drawn with the same icons as in panels A and B for graphical consistency, but it was not measured in this model. The arrows from EGFR to PI3K/Akt and MEK/ERK are drawn solid to keep the receptor-to-kinase relationship legible; EGFR dependence was likewise not tested in this model, and only the kinase activation and the reduction in cell death were measured. Symbols as in Figure 1. EGFR, epidermal growth factor receptor; ERK, extracellular signal-regulated kinase; GLUT1, glucose transporter 1; GSK-3β, glycogen synthase kinase 3β; HIF-1α, hypoxia-inducible factor 1α; LacCer, lactosylceramide; MEK, mitogen-activated protein kinase kinase; mPTP, mitochondrial permeability transition pore; PI3K, phosphoinositide 3-kinase; RISK, reperfusion injury salvage kinase; VEGF, vascular endothelial growth factor. Created in BioRender. Foglio, E. (2026) https://BioRender.com/rff03rh.

These data support a stage-dependent GM3–EGFR model: excessive GM3 during early commitment suppresses survival signals, whereas controlled conversion of GM3 to LacCer permits progression through the myogenic program. Because EGFR signaling must subsequently decline for terminal differentiation, neither persistent NEU3 overexpression nor persistent EGFR activity should be assumed to be uniformly beneficial.

NEU3 therefore acts as a temporal gatekeeper rather than a simple differentiation switch, tuning ganglioside composition to match the signaling needs of successive myogenic stages (Fig. 2A).

NEU3 overexpression in C2C12 cells can delay fusion while ultimately producing hypertrophic myotubes with larger diameters and more myonuclei, consistent with sustained PI3K/Akt/mTOR signaling (52). This observation illustrates the importance of separating short-term pathway activation from the final differentiation phenotype.

In myoblasts from older individuals, DMOG-mediated stabilization of HIF signaling partly restores myogenic markers (53). A role for NEU3 in that rescue is plausible because NEU3 and HIF-1α can participate in a feedback circuit (32), but it has not been demonstrated. Direct NEU3 activation as a therapy for sarcopenia therefore remains a hypothesis rather than an established strategy.

Ganglioside remodeling during myogenesis extends beyond total GM3 abundance and includes changes in individual GM3 molecular species (54). Comparable evidence is not available for GD3, so the discussion and Figure 2 are confined to the GM3-to-LacCer transition 45, 50, 54.

### Hypoxia and cardioprotection

A further context is NEU3’s role in hypoxic adaptation. Ischemia and low oxygen are common stressors, and adaptation is governed chiefly by the master regulator hypoxia-inducible factor 1α (HIF-1α).

Hypoxia-induced NEU3 expression has been linked to Sp1/Sp3 in C2C12 cells (32); the promoter study itself contains no hypoxia experiment (31). Evidence that hypoxia-responsive and Sp-family pathways converge on other target genes provides context for this promoter architecture but is not NEU3-specific (34); the two levels of evidence should not be conflated.

Cell studies support a feed-forward model in which hypoxia increases NEU3, conversion of GM3 to LacCer reduces ganglioside-mediated restraint of EGFR, and ligand-responsive EGFR signaling through PI3K/Akt and ERK reinforces HIF-1α accumulation 32, 55, 56, 57, 58, 59. NEU3 silencing attenuates this response and increases apoptosis, whereas pharmacological inhibition of EGFR or PI3K reduces the protection associated with NEU3 overexpression (32).

This proposed hypoxia–Sp1/Sp3–NEU3–EGFR–HIF-1α circuit can reinforce survival programs, including the glycolytic and angiogenic responses shown in Figure 2B. That panel shows only the hypoxic stimulus upstream of NEU3 induction and omits the Sp1/Sp3-dependent transcriptional step described above. Its operation and strength are likely to differ with ligand availability, receptor abundance, and duration of hypoxia; the available data do not justify assuming autonomous EGFR activation in every NEU3-high cell.

In right-atrial appendage samples from children with cyanotic congenital heart disease, NEU3 abundance correlated with HIF-1α and phosphorylated EGFR and increased with hypoxia severity relative to acyanotic controls (60).

These human data are consistent with engagement of the NEU3–EGFR–HIF-1α axis during chronic myocardial hypoxia, but their observational design does not by itself prove cardioprotection or establish causal direction.

In H9c2 cardiomyoblasts exposed to simulated ischemia–reperfusion injury, NEU3 overexpression reduced cell death and lactate dehydrogenase release and sustained HIF-1α (61). The protective phenotype depended on PI3K and MEK activity, linking NEU3 to the reperfusion injury salvage kinase (RISK) network (Fig. 2C). That study measured NEU3 activity, Akt and ERK phosphorylation, HIF-1α and cell death; GM3-to-LacCer conversion, EGFR dependence, GSK-3β and mitochondrial permeability transition pore (mPTP) control were not assayed in the same experiment and are carried here as pathway context.

The RISK network comprises PI3K/Akt and MEK/ERK arms that can converge on GSK-3β, endothelial nitric-oxide synthase, and mPTP control (62). NEU3 is best positioned upstream as a ganglioside-dependent sensitizer of receptor signaling rather than as a direct component of the mitochondrial machinery.

The model proposed on the basis of these data is that NEU3 converts GM3 to LacCer and enhances EGFR-dependent survival signaling, thereby raising RISK-pathway activity during simulated injury; the ganglioside and receptor steps of that chain remain inference rather than measurement (61).

These observations nominate transient NEU3 modulation as a testable cardioprotective strategy, but evidence is currently preclinical and relies substantially on overexpression in a cardiomyoblast model. Independent in vivo target-engagement and safety studies are required, particularly because sustained NEU3 activity can have opposing effects.

### Ganglioside catabolism and storage disorders

NEU3 provides alternative catabolic routes in ganglioside storage disorders. Desialylation of GM2 to GA2 can partially bypass hexosaminidase deficiency in mouse models 63, 64, 65, while desialylation of GM1a to GA1 reduces GM1a burden in experimental GM1 gangliosidosis (43). Shotgun lipidomics of *Hexa*^−/−^*Neu3*^−/−^ brain further shows secondary glycerophospholipid and sphingomyelin changes together with demyelination and axonal degeneration, indicating that loss of the Neu3 bypass has consequences beyond GM2 accumulation itself (66). Independently, human neuronal models of GM2 gangliosidosis show that the plasma membrane itself is altered in lipid and protein composition, in part through lysosomal exocytosis, with measurable effects on synaptic protein abundance and neuronal activity, indicating that the compartment in which NEU3 acts is itself remodeled by the disease (67). In the 2026 Tay–Sachs study, intrathecal AAVrh10-hNEU3 was evaluated alone and with istradefylline in *Hexa*^−/−^*Neu3*^−/−^ mice; the reported reductions in GM2 burden and the improvements in neuropathological, body-weight, survival, and motor readouts were obtained with the combined regimen, and efficacy of AAV-hNEU3 as monotherapy was not established (64). These findings strengthen causal evidence in vivo while remaining preclinical.

Species differences are central: endogenous Neu3-dependent bypass helps explain why some mouse models are milder than human disease, and combined *Hexa/Neu3* models are being developed to improve translational fidelity 63, 65. NEU3 replacement is therefore promising, but dose, tissue distribution, immunogenicity, and off-target ganglioside remodeling must be established before clinical extrapolation.

## Metabolic and immune contexts

### Insulin signaling

NEU3’s role in metabolism is complex, placing it at the intersection of lipid signaling and insulin action. The insulin receptor is heavily glycosylated, and its signaling depends on the ganglioside composition of its membrane microenvironment (68).

GM3 is a potent negative regulator of insulin signaling. Elevated GM3, often from TNF-α-driven GM3 synthase upregulation in inflammation and obesity, impairs insulin-receptor activation by disrupting the insulin receptor–caveolin-1 association and dissociating the receptor from its signaling platform, causing GM3-dependent insulin resistance 68, 69.

The relationship between NEU3 and insulin signaling is context dependent. GM3 accumulation can impair insulin-receptor organization in caveolar microdomains, and mice lacking GM3 synthase show enhanced insulin sensitivity 69, 70. In the opposite direction, mice overexpressing human NEU3 develop a diabetic phenotype by 18–22 weeks, with hyperinsulinemia, islet hyperplasia, increased β-cell mass, and reduced insulin-stimulated phosphorylation of the insulin receptor and IRS1 in muscle (71). Conversely, olanzapine induced a sialidase activity attributed to NEU3 in NEU1-deficient human sialidosis fibroblasts, and in separate experiments on wild-type human fibroblasts olanzapine reduced insulin-stimulated IGF-receptor and IRS1 phosphorylation; the assignment to NEU3 rests on sensitivity to DANA, itself not a NEU3-selective inhibitor, and to anti-NEU3 antibodies, measured in a third system, rather than on isoenzyme-resolved catalytic measurement (72). Desialylation of insulin-receptor N-glycans can likewise lower receptor autophosphorylation in adipocytes, although in that system the activity was attributed to the NEU1-containing elastin receptor complex rather than to NEU3 (73), a reminder of how readily receptor desialylation is assigned to the wrong isoenzyme. These findings argue against a simple model in which lowering GM3 uniformly improves insulin responsiveness and instead point to tissue-specific effects of ganglioside composition and receptor desialylation.

### Immune cells and inflammation

Gangliosides are present in immune-cell membrane microdomains, where composition varies with lineage and activation state, although the nervous system carries by far the highest ganglioside abundance; within these microdomains they influence receptor clustering. CD4 and CD8 T cells require different ganglioside repertoires for efficient activation (74), and tumor-derived gangliosides can recruit and support myeloid-derived suppressor cells (75). Extracellular NEU3 can also prime freshly isolated human neutrophils in vitro (76), and native NEU1 and NEU3 activity modulates CD22 cluster size, lateral mobility, and calcium responses in human B cells (77). In rheumatoid arthritis, erythropoietin drives NEU3 upregulation through signal transducer and activator of transcription 5 in fibroblast-like synoviocytes, linking NEU3 induction to desialylation in a human autoimmune disease (78). These findings establish that ganglioside remodeling can shape immunity, but direct causal links between tumor-cell NEU3 and immune evasion remain to be tested.

## Pathological roles

Persistent or mislocalized NEU3 activity is associated with malignancy, fibrosis, and intestinal inflammation. The evidence varies from mechanistic cell experiments to causal knockout studies, and not every disease-linked increase in sialidase activity has been assigned unambiguously to NEU3.

### Cancer

NEU3 expression or activity is elevated in multiple cancer models and selected human tumors, with the strongest mechanistic literature in colorectal and renal cell carcinoma 35, 79, 80, 81; NEU3 is also overexpressed in human bladder-cancer tissue and cell lines, where silencing reduces invasion together with ERK and PI3K signaling (82). In gastric cancer, by contrast, NEU3 immunoreactivity is markedly higher in tumor than in non-tumoral tissue yet shows no association with Lauren classification, angiolymphatic invasion, or histological grade (83). Across these systems, increased NEU3 can reduce pro-apoptotic ganglioside signals and modify receptor or adhesion-complex organization. Evidence for prognostic value is tumor-specific and should not be generalized across all malignancies.

These mechanisms share a theme: remodeling the membrane ganglioside landscape so that pro-survival and pro-proliferative signaling can be enhanced.

In colorectal cancer, NEU3 upregulation suppresses apoptosis through Akt-associated signaling (35). Notably, NEU3 overexpression enhances EGFR activation only in cells that already express EGFR above a threshold, and this enhancement is counterbalanced by cetuximab (84), which illustrates at the level of a single pathway that the NEU3 effect is conditional on receptor context rather than absolute. Cell studies also connect NEU3 to Wnt/β-catenin signaling through LRP6 and to stem-like phenotypes (85). These pathways may cooperate, but the relative contribution of direct glycoprotein desialylation versus local ganglioside remodeling is not fully resolved (Fig. 3A).

**Fig. 3:**
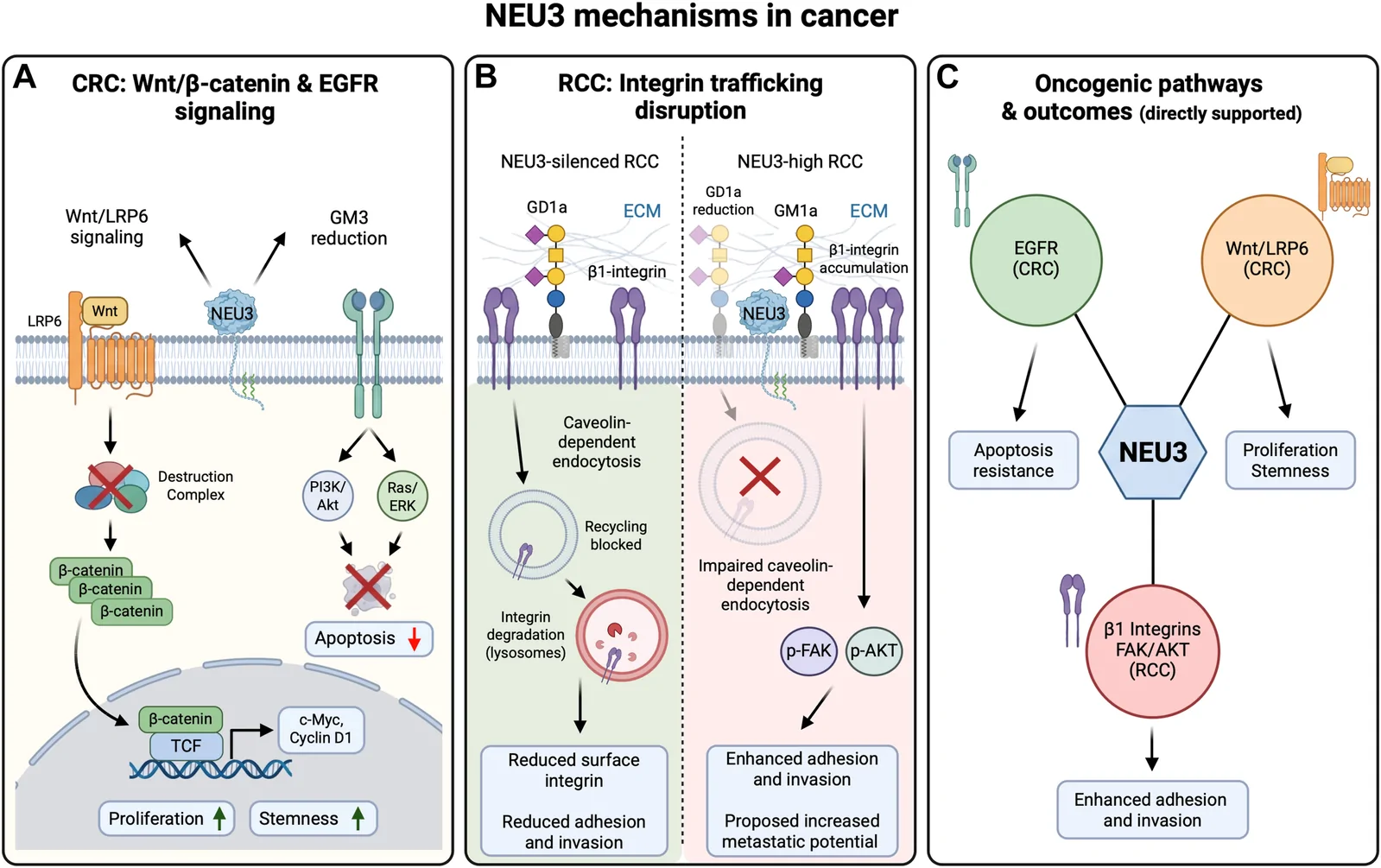
NEU3 mechanisms in cancer. A, CRC: Wnt/β-catenin and EGFR signaling. NEU3 lowers GM3 and enhances LRP6 phosphorylation, which the panel labels Wnt/LRP6 signaling; impaired assembly of the β-catenin destruction complex permits nuclear β-catenin–TCF transcription of c-Myc and cyclin D1 with increased proliferation and stem-like traits, while the accompanying reduction in GM3 relieves a ganglioside brake on EGFR-dependent PI3K/Akt and Ras/ERK signaling and reduces apoptosis. LRP6 glycans are not drawn: the cited study attributes the effect to ganglioside modulation, and desialylation of LRP6 by a human sialidase has not been tested. The seven-transmembrane receptor drawn beside LRP6 is Frizzled, the Wnt receptor with which LRP6 acts as co-receptor. B, RCC: integrin trafficking disruption. Both states are RCC cells. In the NEU3-silenced state GD1a accumulates, β1 integrin is internalized by caveolar endocytosis, its recycling is blocked and it is routed to lysosomes, lowering surface integrin and giving reduced adhesion and invasion. In the NEU3-high state GD1a is converted to GM1a by removal of its terminal sialic acid, caveolar endocytosis of β1 integrin is reduced, surface integrin is maintained, and p-FAK and p-Akt signaling increases, with enhanced adhesion and invasion in vitro. The panel labels the downstream consequence a proposed increase in metastatic potential; metastasis itself was not assessed in the cited work, and the inference is drawn from the in vitro phenotype alone. No normal-kidney baseline is implied, and none is drawn. β1 integrin is shown as part of an αβ1 heterodimer; the α subunit is not specified. C, Oncogenic pathways and outcomes (directly supported). Only receptor pathways with direct support in the tumor contexts discussed are shown: EGFR in colorectal models, Wnt/LRP6 in colorectal cancer, and β1 integrin–FAK/Akt in renal cell carcinoma. The breast and ovarian observations in those series report NEU3-dependent survival and expression associations without establishing a receptor mechanism in those tissues, the prostate evidence concerns androgen-receptor signaling, and pathways without direct support in these models, including c-Met, are not shown. Glycan symbols follow SNFG as in Figure 1, with a yellow square for GalNAc in GD1a and GM1a. In the colorectal panel GM3 reduction is named rather than drawn, and its downstream consequence is shown; the GM3-to-LacCer conversion itself is depicted in Figures 1 and 2. NEU3 is shown with the same non-canonical membrane association throughout. Akt, protein kinase B; CRC, colorectal cancer; ECM, extracellular matrix; EGFR, epidermal growth factor receptor; ERK, extracellular signal-regulated kinase; FAK, focal adhesion kinase; GalNAc, N-acetylgalactosamine; GD1a, disialoganglioside with one sialic acid on each of the inner and terminal galactose residues; GM1a, monosialotetrahexosylganglioside, the product of terminal desialylation of GD1a; GM3, monosialodihexosylganglioside; LRP6, low-density lipoprotein receptor-related protein 6; PI3K, phosphoinositide 3-kinase; RCC, renal cell carcinoma; TCF, T-cell factor; SNFG, Symbol Nomenclature for Glycans. Created in BioRender. Foglio, E. (2026) https://BioRender.com/ndzsvby.

NEU3 also amplifies Wnt/β-catenin signaling, a primary CRC driver. Canonical Wnt requires Wnt binding to Frizzled and the co-receptor LRP6. NEU3 enhances LRP6 phosphorylation and accelerates the recruitment of Axin and GSK-3β to the receptor, and catalytically inactive NEU3 does not, so the effect requires catalysis. The authors attribute it to ganglioside modulation rather than to desialylation of LRP6 itself; no study has tested whether LRP6 N-glycans are substrates of any human sialidase, so the two possibilities have not been separated experimentally (85). The consequence is impaired assembly of the β-catenin destruction complex and increased nuclear β-catenin, which acts on canonical Wnt target genes such as c-Myc and cyclin D1. NEU3 also sustains stem-like traits in colon cancer cells, including clonogenicity and sphere formation (85).

In renal cell carcinoma, NEU3-dependent ganglioside remodeling alters β1 integrin trafficking and adhesion (86). This mechanism provides a receptor-independent example of how membrane lipids can influence invasive behavior.

The comparison that supports this mechanism was made within RCC cells, not against normal kidney. Silencing NEU3 in RCC cells raised membrane ganglioside content, GD1a in particular, enhanced caveolar endocytosis of β1 integrin and blocked its recycling, lowered surface integrin, and reduced EGFR and FAK/Akt signaling, with decreased adhesion and decreased invasiveness in vitro; the NEU3-high state shows the converse (86) (Fig. 3B). Human tissue data do not support a GD1a-poor tumor relative to normal kidney: a-series gangliosides including GD1a have been reported as higher in renal cell carcinoma than in normal kidney (87). The mechanism should therefore be read as a within-tumor dependence on NEU3 activity rather than as a normal-versus-tumor contrast.

NEU3 is also elevated in prostate-cancer models, where it has been linked to proliferation and reduced sensitivity to androgen deprivation 81, 88. Receptor pathways that lack direct support in these prostate models, including c-Met, are not shown in Figure 3C.

NEU3 silencing induces apoptosis in HeLa cells, with decreased expression of the anti-apoptotic protein Bcl-x_L_ and increased MDA7 and GM3 synthase transcripts, and the colon-carcinoma line HT-29 and the breast-carcinoma line MCF-7 were reported to be similarly affected (79). NEU3 immunoreactivity correlates with the T factor of the pathological tumor-node-metastasis classification in ovarian clear-cell adenocarcinoma (89). Neither study addresses integrin signaling, and neither establishes a receptor mechanism in breast or ovarian tissue. The breadth of these observations supports further study but does not yet establish a single pan-cancer mechanism.

NEU3 silencing also inhibits angiogenic behavior in EA.hy926 endothelial cells through Wnt/β-catenin-associated signaling (90). Together, the cancer and endothelial studies motivate multi-compartment experiments that distinguish tumor-cell-autonomous effects from stromal and vascular contributions (Fig. 3).

Proposed drivers of tumor-associated NEU3 include persistent hypoxia, Sp-family transcriptional input, oncogenic signaling, and genomic or epigenetic changes 5, 10, 31, 32. However, direct evidence is uneven: NEU3 promoter hypomethylation has not been established, and location within a chromosomal region amplified in some tumors does not by itself demonstrate NEU3 amplification. These mechanisms should therefore be tested rather than assumed.

### Fibrosis

NEU3 has emerged as a profibrotic regulator in lung and liver models. In addition to membrane ganglioside remodeling, extracellular NEU3 has been proposed to increase activation of latent TGF-β1 (Fig. 4) 36, 37, 91, 92. The direction of this relationship is not uniform across tissues: in primary human cardiac fibroblasts, induced NEU3 upregulation lowers GM3 content and instead attenuates TGF-β signaling and collagen I deposition (93), context dependence consistent with the rheostat model developed here.

**Fig. 4:**
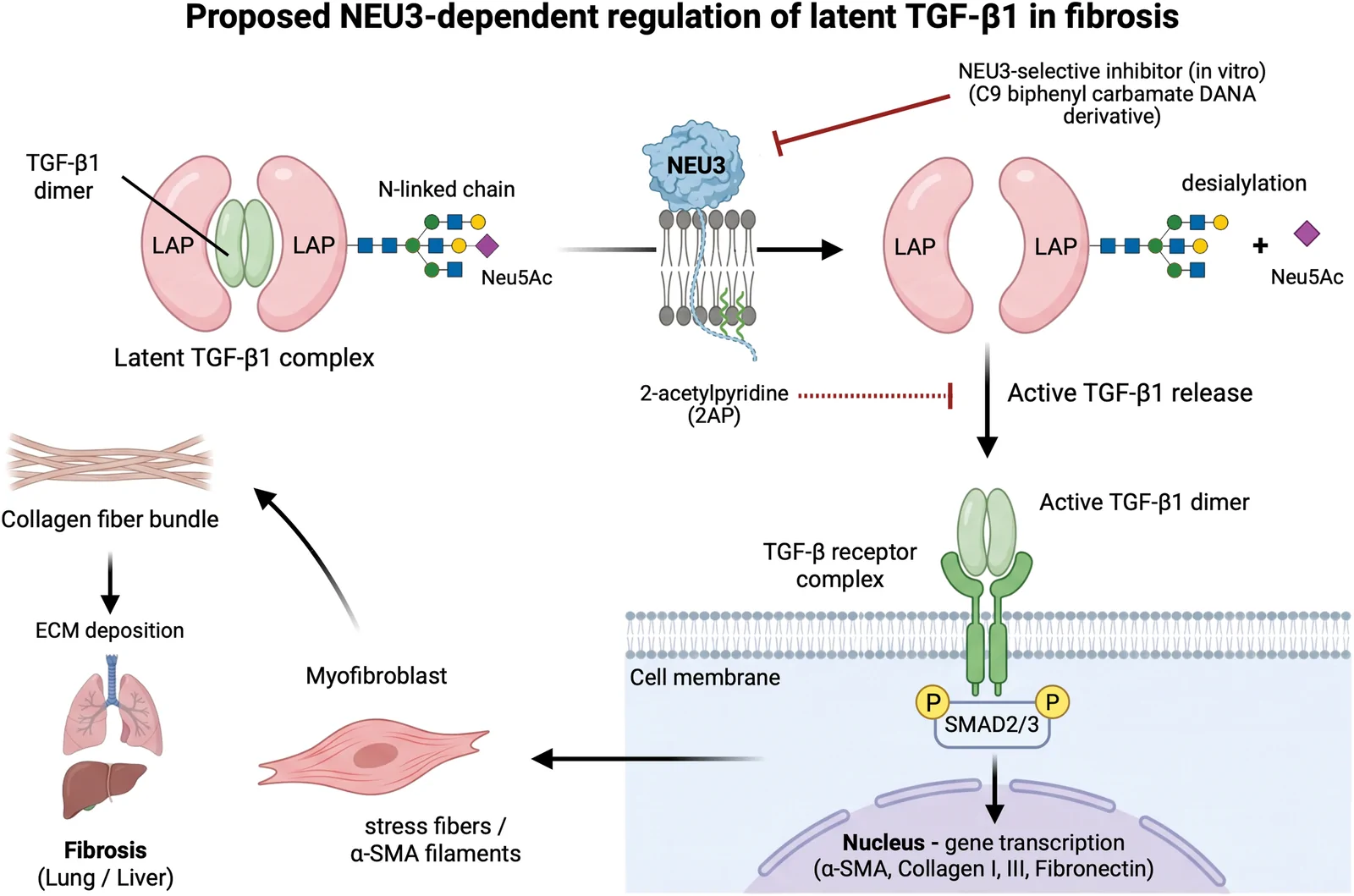
Proposed NEU3-dependent regulation of latent TGF-β1 in fibrosis. The latent complex is drawn as the TGF-β1 dimer enclosed by the latency-associated peptide, each subunit of which carries N-linked chains terminating in Neu5Ac; a single chain is drawn for clarity, and the latent TGF-β-binding protein and its matrix tethering are omitted. Recombinant human NEU3 desialylates these chains and thereby releases active TGF-β1 in a defined biochemical assay; in the cellular sequence drawn here the extracellular pool destabilizes the complex and releases the active TGF-β1 dimer, which engages the TGF-β receptor complex and phosphorylated SMAD2/3 and drives transcription of α-SMA, collagen I and III, and fibronectin, myofibroblast differentiation with stress-fiber and α-SMA assembly, collagen-fiber and extracellular-matrix deposition, and fibrosis in lung and liver. The panel labels the enzymatic step simply desialylation, because it is demonstrated biochemically; the cellular sequence around it is what remains proposed, since the step has not been shown on endogenous latent TGF-β1 in tissue and the available studies do not establish that a defined number of terminal sialic acids is individually required for the integrity of the latent complex. NEU3 is drawn in a membrane because the enzyme is membrane-associated; the pool acting on the secreted latent complex is extracellular, and vesicle-associated release is one route by which an enzyme without a signal peptide may reach it 39, 40, while whether a soluble extracellular form exists is unresolved. Glycan symbols follow SNFG as in Figure 1, with green circle, Man and blue square, GlcNAc. The two pharmacological agents are drawn separately because the evidence behind them differs in kind rather than in strength. The C9 biphenyl carbamate DANA derivative is the most selective NEU3 inhibitor reported in biochemical assays against the four human isoenzymes, but it has not been tested in any fibrosis model, and it is shown acting on the enzyme. 2AP inhibits NEU3-mediated release of active TGF-β1 with nanomolar potency and is antifibrotic in vivo, but its isoenzyme selectivity has not been demonstrated, so it is drawn as an inhibitory bar on that release step rather than on the enzyme itself. Neither compound has shown in vivo target engagement. C9-BA-DANA is not shown, because it is a NEU1-selective inhibitor rather than a NEU3 probe. 2AP, 2-acetylpyridine; DANA, 2,3-didehydro-2-deoxy-N-acetylneuraminic acid; ECM, extracellular matrix; GlcNAc, N-acetylglucosamine; Man, mannose; SMAD, small mothers against decapentaplegic homolog; α-SMA, α-smooth muscle actin; TGF-β1, transforming growth factor β1; SNFG, Symbol Nomenclature for Glycans. Created in BioRender. Cirillo, F. (2026) https://BioRender.com/yqnro93.

TGF-β1 is secreted in a latent complex in which the mature cytokine is non-covalently associated with the glycosylated latency-associated peptide (LAP); incorporation of latent TGF-β-binding protein can tether this complex to extracellular matrix (94). Activation releases bioavailable TGF-β1 and initiates TGF-β-receptor and SMAD signaling.

LAP is sialylated, and recombinant human NEU3 desialylates LAP and thereby releases active TGF-β1 from the latent complex in a defined biochemical assay (95). Which terminal sialic acids are individually required for the integrity of the latent complex has not been established, and the same step has not been demonstrated on endogenous latent TGF-β1 in tissue; Figure 4 therefore depicts the cellular sequence as proposed.

In bleomycin-induced pulmonary fibrosis, lung Neu3 increases, Neu3 deficiency reduces inflammation and collagen accumulation, and recombinant murine Neu3 worsens disease 36, 37, 96. TGF-β1 itself raises NEU3 protein in human lung epithelial cells without changing NEU3 mRNA abundance, by slowing NEU3 degradation and shifting its transcript from monosomes into polysomes (97), and a recent study further describes a rapid profibrotic positive-feedback loop in which LAP increases NEU3 translation (98). Together, these data support causality in mice while leaving the exact extracellular substrate sequence to be resolved.

The principal causal observations in the pulmonary model are.

(i) Neu3-knockout mice are strongly protected from bleomycin-induced fibrosis, with less inflammation, less accumulation of active TGF-β1, and less collagen than wild-type mice (37).
(ii) Oropharyngeal administration of recombinant murine Neu3 causes both inflammation and fibrosis, indicating that the enzyme is sufficient to induce fibrosis in this model, whereas a catalytically inactive mutant causes inflammation without fibrosis, indicating that catalytic activity is required for the fibrotic arm; when given after bleomycin the effect was seen in male but not female mice, and no equivalent experiment has been performed with human NEU3 (96).
(iii) DANA and oseltamivir, neither of which is NEU3-selective, attenuate fibrosis when given after bleomycin (36), and compounds of the 2-acetylpyridine class, which inhibit NEU3-mediated release of active TGF-β1 with nanomolar potency in vitro, attenuate fibrosis at 0.1–1 mg/kg (95). This supports pharmacological tractability while not, by itself, proving isoenzyme selectivity.

In carbon-tetrachloride-induced liver injury, 2-acetylpyridine (2AP) reduced perivascular inflammation and the accumulation of Mac2-positive macrophages in both sexes, reduced picrosirius red staining in both sexes and liver hydroxyproline in males only, and reversed the tissue desialylation and NEU3 upregulation caused by the injury, without affecting steatosis (91). Because 2AP has not been counter-screened against the other human isoenzymes and no inhibition constant against a canonical sialidase substrate has been reported, we refer to it as a NEU3-directed compound of undemonstrated isoenzyme selectivity. In fibrotic disease the high sialidase activity of the lesion produces a measurable desialylation of serum proteins, which has been proposed as a surrogate biomarker (91). One isoenzyme-resolved measurement does exist in metabolic liver disease: serum NEU3 protein was raised by immunoblot in patients with non-alcoholic fatty liver disease but not in those with steatohepatitis, whereas desialylation of a serum glycoprotein was raised in both (91). Whether total serum sialidase activity in this setting tracks NEU3 has not been tested.

### Intestinal inflammation

In intestinal inflammation, Neu3 acts through a distinct route centered on intestinal alkaline phosphatase (IAP). IAP, a brush-border enzyme on the apical enterocyte surface, dephosphorylates and detoxifies bacterial lipopolysaccharide (LPS), preventing excessive innate immune activation by the commensal microbiota (99).

Yang *et al.* showed that in a mouse model of recurrent *Salmonella enterica* serovar Typhimurium food poisoning, which produces progressive and enduring colitis, Neu3 is induced in the intestinal epithelium and desialylates IAP. Desialylated IAP is rapidly degraded, losing protective activity; LPS detoxification fails at the mucosal barrier, driving TLR4-mediated inflammation and severe colitis (99).

Animal models reinforce this, as Neu3-deficient mice are protected from this cascade. In *Neu3*^−/−^ animals, IAP stays high during infection, enabling LPS detoxification and markedly less colitis than wild-type mice (99). This adds another facet to the destructive face of NEU3: its induction can be harmful in the gut by destroying a key protective enzyme.

## Therapeutic modulation, biomarkers, and evidence limits

The available evidence is better captured by a multidimensional rheostat than by a good-versus-bad dichotomy. Outcome depends on magnitude and duration of activity, subcellular or extracellular localization, ganglioside substrate availability, receptor context, and the capacity of homeostatic feedback to restore baseline.

Transient, spatially restricted NEU3 can support adaptation or differentiation, whereas sustained or ectopic activity can reinforce growth, inflammatory, or fibrotic circuits. These are working principles, not universal rules: each therapeutic proposal must identify the relevant cell type, biochemical substrate, exposure window, and in vivo target-engagement marker.

NEU3 is pharmacologically attractive because it lies upstream of several signaling pathways, but that same position creates risk. Systemic inhibition may interfere with adaptive or catabolic functions, whereas sustained activation may favor tumor or inflammatory signaling. Selectivity, tissue restriction, exposure duration, and direct measurement of ganglioside products are therefore central design requirements.

### Inhibition in fibrosis and cancer

DANA (Neu5Ac2en) inhibits mammalian sialidases but lacks NEU3 isoenzyme selectivity, and anti-influenza drugs such as oseltamivir and zanamivir are not suitable NEU3 probes 100, 101. Active-site models of the human enzymes provide a starting point for selective inhibitor design (38), and the synthetic methodology developed for modified Neu5Ac scaffolds directed at viral neuraminidases offers transferable chemistry, although those enzymes differ substantially from the human isoenzymes in active-site architecture and substrate preference despite the shared β-propeller fold 102, 103, 104, 105, 106, 107.

More selective chemical and genetic tools are now beginning to separate NEU3-dependent biology from total sialidase activity.

The C9 substituent governs which isoenzyme a DANA analogue prefers, and the distinction matters because compounds from this scaffold have been assigned to the wrong enzyme in the secondary literature. A DANA library modified at C4 and C9 yielded inhibitors selective for NEU3, the best with a *K*_*i*_ = 0.32 μM and approximately 15-fold selectivity over the other human isoenzymes (108). By contrast, C9-butyl-amide-DANA (C9-BA-DANA) is an established NEU1-selective inhibitor and is not a NEU3 probe (109). Systematic replacement of the C9 amide with bioisosteric linkers has since yielded a C9 biphenyl carbamate with sub-micromolar potency (*K*_*i*_ = 0.12 μM) and pronounced selectivity for NEU3 over the other human isoenzymes (110), which defines the current benchmark for biochemical NEU3 selectivity; in vivo target engagement has not been demonstrated for any compound in this series. These compounds are valuable probes, although cellular permeability, pharmacokinetics, and tissue distribution remain to be optimized.

The compound 2-acetylpyridine inhibits the release of active TGF-β1 by recombinant human NEU3 with an IC_50_ of about 40 nM, and by the recombinant mouse enzyme with an IC_50_ about 20-fold higher, and it reduces fibrosis in mouse models of pulmonary and hepatic injury 91, 95. The species gap deserves note, because every in vivo experiment reported for this class has been performed in mice. That potency was measured in a coupled functional assay rather than on a canonical sialidase substrate; the compound has not been counter-screened against NEU1, NEU2, or NEU4, no inhibition constant against a canonical sialidase substrate has been reported, and in vivo target engagement has not been shown. It should therefore be described as a NEU3-directed inhibitor of undemonstrated isoenzyme selectivity rather than as a validated selective NEU3 inhibitor.

In cancer, genetic or pharmacological NEU3 suppression may restore ganglioside-mediated restraint of receptor and adhesion signaling, but combination claims involving EGFR inhibitors or chemotherapy remain hypotheses until tested with selective tools in appropriate in vivo models (85).

### Activation and replacement strategies

ST3GAL5 inhibition reduces de novo GM3 synthesis and can phenocopy one metabolic consequence of NEU3 activity; it does not activate NEU3. This substrate-reduction strategy must therefore be separated from direct enzyme activation or delivery. CMP-Neu5Ac-inspired sialyltransferase inhibitors illustrate the concept, but efficacy and safety in NEU3-relevant disease models remain to be established 70, 111.

Intrathecal AAVrh10-hNEU3 combined with istradefylline reduced GM2 burden and improved neuropathological, body-weight, survival, and motor readouts in a Tay–Sachs mouse model; efficacy of AAV-hNEU3 as monotherapy was not established (64). This supports further evaluation of replacement strategies without establishing efficacy of NEU3 monotherapy. Two caveats bear on replacement strategies. The transgene used here is the human enzyme delivered into a Neu3-null host, so the enzymatic component of the rescue observed already reflects human NEU3 activity rather than that of the more efficient mouse ortholog; what remains unknown is the expression level needed to reproduce it in patients. Separately, for strategies that aim to raise endogenous NEU3 rather than to deliver it, the human enzyme is roughly half as efficient as the mouse enzyme at converting GM1a to GA1 (section 2.2), and the corresponding ratio for the GM2-to-GA2 step relevant to Tay–Sachs has not been measured. Recombinant enzyme or tissue-restricted gene delivery for ischemia or muscle regeneration remains more speculative and requires biodistribution, dosing, and oncogenic-safety studies. A conceptually different approach uses NEU3 as a trigger rather than as a target: sialic acid-capped DNA nanostructures are unmasked by tumor NEU3 to expose galactose ligands for asialoglycoprotein-receptor-mediated uptake, confining release of a cytotoxic payload to hepatocellular carcinoma in preclinical models (112).

### Biomarkers and translational limitations

Circulating or tissue NEU3 may prove useful as a diagnostic, prognostic, or pharmacodynamic marker, but total sialidase activity is not NEU3-specific. Assays should combine isoenzyme-resolved protein or activity measurements with substrate/product lipidomics and must be validated prospectively in independent cohorts. Human cohort data are beginning to appear: in a Bangladeshi birth cohort of 189 children, total fecal NEU3 activity at 15 months was inversely associated with length-for-age z-scores at 18, 21, and 24 months, with small effect sizes (113), a finding consistent with the intestinal alkaline phosphatase mechanism discussed above; and in a prospective cohort of 194 couples undergoing in vitro fertilization, spermatozoal NEU3 abundance was associated with fertilization rate within the subgroup having at least 80% metaphase II oocytes and normal semen analysis, with modest discrimination (area under the curve 0.67); spermatozoal NEU1 performed equivalently in the same cohort, so the finding does not by itself establish NEU3 specificity (114). These are among the first observations linking NEU3 to a clinical outcome in humans, and together they illustrate the present limits of the approach: one measures enzymatic activity and the other protein abundance, in different tissues, with no shared assay standard.

The main translational opportunities are selective inhibition in fibrosis or selected cancers, replacement in defined ganglioside storage disorders, and biomarker-guided target engagement. All remain preclinical or investigational. Substrate reduction by ST3GAL5 inhibition is a strategy distinct from direct NEU3 modulation, and Figure 5B shows the two separately.

**Fig. 5:**
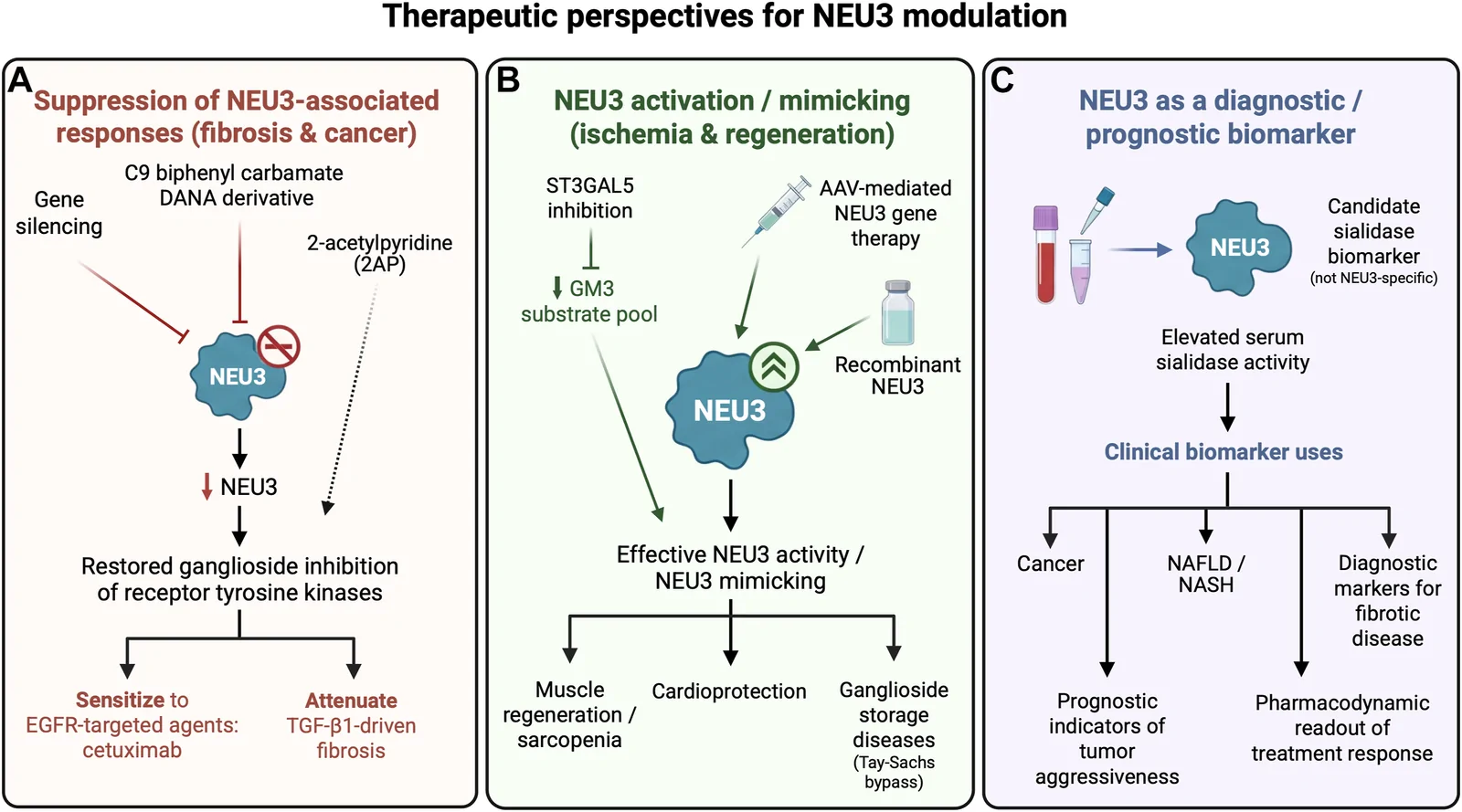
Therapeutic perspectives for NEU3 modulation. A, suppression of NEU3-associated responses (fibrosis and cancer): a direct-enzyme branch groups gene silencing with the C9 biphenyl carbamate DANA derivative, the most potent and selective NEU3 inhibitor reported from this scaffold to date, whereas 2-acetylpyridine (2AP) is routed separately to NEU3-associated functional readouts because its isoenzyme selectivity has not been demonstrated: it has not been counter-screened against NEU1, NEU2, or NEU4, and its reported potency comes from a coupled functional assay rather than from a canonical sialidase substrate. C9-BA-DANA is not shown, because it is NEU1-selective. No compound shown has demonstrated NEU3 target engagement in vivo. The consequence is restored ganglioside inhibition of receptor tyrosine kinases, with the aims of sensitizing tumors to EGFR-targeted agents such as cetuximab and of attenuating TGF-β1-driven fibrosis. B, NEU3 activation or mimicking (ischemia and regeneration): AAV-mediated gene therapy and recombinant enzyme raise NEU3 activity, whereas ST3GAL5 inhibition does not act on the enzyme but lowers the GM3 substrate pool and therefore mimics one metabolic consequence of NEU3 activity; candidate settings are muscle regeneration and sarcopenia, cardioprotection, and ganglioside storage disorders, including bypass of the hexosaminidase block in Tay–Sachs disease. C, NEU3 as a diagnostic or prognostic biomarker: serum protein desialylation has been proposed as a surrogate readout in fibrotic disease and, more speculatively, in metabolic liver disease, labeled in the panel as NAFLD and NASH, the terms used by the cited study, and as a prognostic or pharmacodynamic measure in cancer. The panel labels it a candidate sialidase biomarker that is not NEU3-specific, because total activity does not resolve the isoenzyme; an isoenzyme-resolved NEU3 measurement combined with substrate/product lipidomics is what the text calls for. All applications shown are preclinical or investigational. This figure summarizes strategies rather than membrane events, so NEU3 appears as a schematic enzyme symbol without the membrane topology drawn in Fig. 1, Fig. 2, Fig. 3, Fig. 4. The panel uses no glycan symbols; GM3 appears only as a named substrate pool. AAV, adeno-associated virus; NAFLD, non-alcoholic fatty liver disease; NASH, non-alcoholic steatohepatitis; ST3GAL5, ST3 β-galactoside α-2,3-sialyltransferase 5; other abbreviations as in Figures 1 and 4. Created in BioRender. Cirillo, F. (2026) https://BioRender.com/w44tz6l.

## Conclusions and unresolved questions

NEU3 is best understood as a context-dependent regulator of membrane gangliosides and receptor-proximal signaling. Cell, animal, and human-tissue studies support roles in myogenesis, hypoxic adaptation, cardioprotection, ganglioside catabolism, cancer, fibrosis, and intestinal inflammation, but the strength and direction of effect vary substantially among settings.

At the same time, the field still contains important boundaries that should be stated explicitly. Much of the mechanistic evidence derives from cell culture systems, overexpression models, or selected animal studies, and the strength of evidence is not yet uniform across tissues or disease contexts. In particular, claims of necessity versus contribution should be distinguished carefully, and therapeutic extrapolation remains preliminary until supported by more selective pharmacological tools, conditional genetic models, and broader in vivo validation.

The rheostat model remains useful if it is treated as a testable framework: NEU3 changes local ganglioside composition and thereby alters signaling thresholds, while receptor identity, ligand abundance, membrane organization, cell state, and exposure duration determine the phenotype.

Several key questions remain for future investigation.

(1) Structure, topology, membrane binding, and export: an experimental NEU3 structure, orientation-resolved membrane studies, and direct analysis of biosynthetic trafficking, membrane insertion, and extracellular-vesicle release are needed to explain how NEU3 reaches membrane and extracellular pools and to define substrate access.
(2) Conditional models: tissue-specific and inducible Neu3 loss- and gain-of-function models are required to distinguish developmental compensation from acute, cell-autonomous effects in heart, muscle, immune cells, gut, tumor, and stroma.
(3) Substrates and interactors: spatial lipidomics, glycoproteomics, and proximity-based interactomics should map the gangliosides, glycoproteins, and membrane complexes modified by endogenous NEU3 in each tissue.
(4) Immune modulation: studies should test whether NEU3 directly controls T-cell, myeloid-cell, or neutrophil function in vivo and whether tumor-cell and host-cell NEU3 have distinct effects on antitumor immunity.
(5) Pharmacology and biomarkers: catalytic binding, isoenzyme profiling, substrate-to-product lipidomics, pharmacokinetics, and tissue target engagement are essential. In particular, 2AP should not be treated as an isoenzyme-selective inhibitor until it has been counter-screened against NEU1, NEU2, and NEU4 and in vivo target engagement has been shown.
(6) Redox control: Hata *et al.* reported that free sulfhydryl groups are required for the activity of the membrane-associated ganglioside sialidase purified from bovine brain (30), but no direct evidence currently shows that extracellular glutathione, hypoxia, or another redox system regulates NEU3 in vivo. Whether surface-exposed thiols modulate NEU3 activity should therefore be tested directly.

Translation will therefore require precise, time- and tissue-restricted modulation rather than global activation or suppression. That standard should allow NEU3 biology to be tested rigorously while preserving the therapeutic promise suggested by current preclinical studies.

## Data Availability

No new data were created or analyzed in this review. All information discussed is contained within the article and its cited sources.

## Supplemental data

This article contains supplemental data.

## Conflict of interest

The authors declare that they have no conflicts of interest with the contents of this article.
